# Rare Association of Bilateral Central Salzmann Nodular Degeneration and Fuchs Endothelial Dystrophy Managed by DMEK: A Case Report

**DOI:** 10.3390/life16030406

**Published:** 2026-03-03

**Authors:** Alina Gabriela Gheorghe, Ana Maria Arghirescu, Maria Cristina Marinescu, Doina Mihaela Pop, Liliana Mary Voinea, Radu Ciuluvică

**Affiliations:** 1 Department of Ophthalmology, Clinical Institute of Ophthalmological Emergencies “Prof. Dr. Mircea Olteanu”, 010464 Bucharest, Romania; alina.gheorghe.g@gmail.com; 2 Department of Ophthalmology, Carol Davila University of Medicine and Pharmacy, 020021 Bucharest, Romania; 3 Doctoral School, Carol Davila University of Medicine and Pharmacy, 020021 Bucharest, Romania; 4 Medical Physiology Discipline, Carol Davila University of Medicine and Pharmacy, 020021 Bucharest, Romania; 5 Foisor Orthopaedic Hospital, 030167 Bucharest, Romania; 6 Department of Anatomy, Faculty of Dental Medicine, Carol Davila University of Medicine and Pharmacy, 020021 Bucharest, Romania

**Keywords:** Salzmann nodular degeneration, Salzmann corneal degeneration, DMEK, in vivo confocal microscopy, anterior segment optical coherence tomography, corneal transplant

## Abstract

Salzmann nodular degeneration is a slowly progressive, rare, degenerative disease of the cornea, usually associated with other ocular pathologies such as dry eye disease, previous ocular surgery, involving the peripheral cornea. We present the surgical management of a rare case of a patient diagnosed with Fuchs endothelial dystrophy where the Salzmann nodules present an unusual bilateral central corneal involvement and overlap the area with the most pronounced endothelial dysfunction. The patient underwent anterior superficial keratectomy, cataract surgery and Descemet membrane endothelial keratoplasty (DMEK). In vivo confocal microscopy and anterior segment optical coherence tomography have been used to monitor the morphological and structural evolution.

## 1. Introduction

Salzmann nodular degeneration (SND) is a progressive, degenerative, rare disease of the cornea, first described in 1925 [1], usually associated with other ocular or systemic pathologies characterized by elevated, whitish-gray subepithelial nodules located anterior to a disrupted Bowmann membrane and composed histologically of fibrotic extracellular matrix. The lesions are typically peripheral or mid-peripheral and may induce visual impairment when they encroach upon the visual axis [2]. Most available epidemiologic data derive from tertiary cornea clinic case series. In a large clinic-based cohort, Paranjpe et al. reported a point prevalence of approximately 1 in 2400 patients (per-patient prevalence) within a tertiary referral population, with a marked female predominance and a bimodal age distribution peaking in the fifth and eighth decades. Similarly, Farjo et al. and Hamada et al. [3,4], in surgical and referral cohorts (analyzed per patient, with some reports also describing per-eye involvement), confirmed female predominance and typical presentation in middle-aged to elderly individuals. Because these data originate primarily from referral centers, the true population prevalence remains uncertain and SND may be underrecognized or misclassified in general practice settings [3,4,5]. SND is most frequently associated with corneal injury due to previous ocular surgery, trauma or ocular surface disease. Several cases have been described in the literature: poor wound healing after cataract surgery [6], after LASIK [7,8], posterior keratoconus and previous ocular trauma [9], meibomian gland dysfunction [10].

Fuchs endothelial dystrophy (FECD) is a slowly progressive, bilateral, usually inherited disease of the corneal endothelium that progressively leads to the alteration of the endothelial cell and Descemet membrane morphology and function, in advanced cases ultimately leading to corneal endothelial dysfunction and corneal decompensation [11]. Multiple studies have identified corneal modification in the context of Fuchs endothelial dystrophy such as altered corneal nerve density [12], altered keratocyte density with anterior keratocyte depletion [13]. As the endothelial pump fails, fluid accumulates in the stroma and migrates anteriorly, causing subepithelial bullae and chronic epithelial dysfunction. Persistent epithelial edema and subepithelial bullae result in recurrent central corneal erosions. These episodes participate in chronic epithelial dysfunction and can cause physical defects in the Bowman membrane [14,15]. In our case, there is data to support that the endothelial decompensation was one of the determining factors that led to the central dysfunction of the cornea, with recurrent epithelial defects that participated in chronic epithelial dysfunction and Bowmann membrane (BM) defects.

Our case describes an atypical bilateral central localization of the Salzmann nodules, involving the optical zone, directly overlying the area with the most pronounced corneal dysfunction due to the FECD. To our knowledge, we found no other cases detailed in the literature describing bilateral central disposition of SND associated with FECD. In the case presented by Aljindan M.Y. et al. in 2021, the central SND was present unilaterally and they do not mention any association with any preexisting ocular or systemic diseases in their patient [16]. What is more, our case provides insights into how the recovery of the endothelial function by DMEK impacts the rate of recurrence and corneal remodeling.

## 2. Materials and Methods

This is a retrospective case report. The patient underwent a full ophthalmological examination. In vivo confocal microscopy (IVCM) (Heidelberg HRT 3 Rostock Corneal module. Heidelberg Engineering, Heidelberg, Germany) and anterior segment optical coherence tomography (AS-OCT) (CSO MS-39) was performed at the initial presentation and during the follow-ups. Visual recovery was achieved through a series of surgical procedures: superficial keratectomy, cataract surgery with single-piece intraocular lens implantation and Descemet membrane endothelial keratoplasty (DMEK). The patient offered informed written consent and the Ethics Committee of the Ophthalmological Emergencies Institute “Prof. Dr. Mircea Olteanu” approved, with no 3391/12.08.2025.

## 3. Case Report

The patient, a 70 year old woman, without any known systemic diseases, has begun having the following symptoms 3 years prior to the presentation in our clinic: halos around lights, decrease in color contrast, followed by a progressive decrease in visual acuity. During this time, multiple episodes of recurrent central corneal erosions have been described. HSV keratitis cytology and serology have been repeated multiple times, with negative results. The patient has not used any ocular surface lubricants on a regular basis in between the recurrent epithelial erosions episodes. No other ocular infections, inflammatory episodes, blepharitis, conjunctivitis, or dry eye were identified. The patient has never worn contact lenses. No other systemic diseases were identified. At the presentation in our clinic, the best-corrected visual acuity (BCVA) for the right eye (RE) was 20/200 and the left eye (LE) was 20/400.

### 3.1. Preoperative Assessment

**Figure 1:**
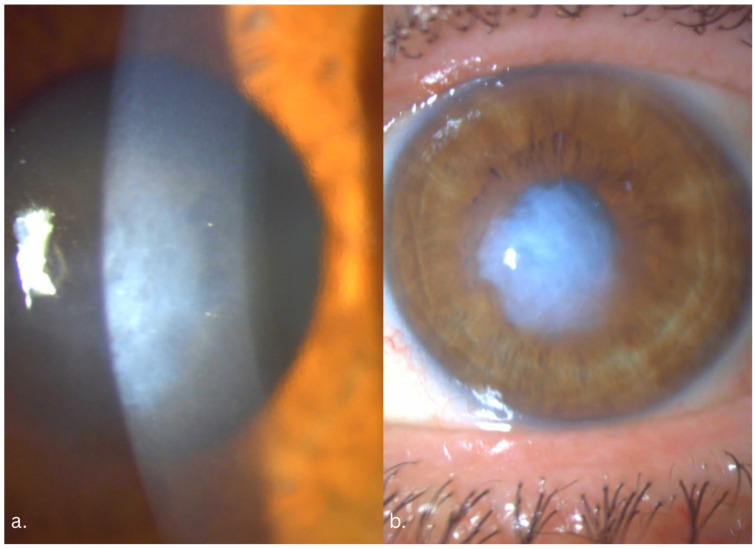
Preoperative slit lamp aspects; (**a**) right eye: central white-gray subepithelial deposits, underlying stromal edema, thickened Descemet membrane, guttae; (**b**) left eye: extensive elevated central subepithelial deposit, predominantly central stromal haze, Descemet membrane folds, guttae.

At presentation, the slit-lamp exam revealed bilateral central white subepithelial elevated opacities, suggestive of Salzmann nodular degeneration, more advanced in the left eye. Underneath the nodule, the stroma was thickened, with mild central stromal haze. Directly underneath the nodule we observed confluent corneal guttae with a thickened Descemet membrane. In the LE, the central endothelium was impossible to visualize due to the advanced central opacity. Peripheral guttae were visible in both eyes (Figure 1). Both eyes presented advanced corticonuclear cataract.

**Figure 2:**
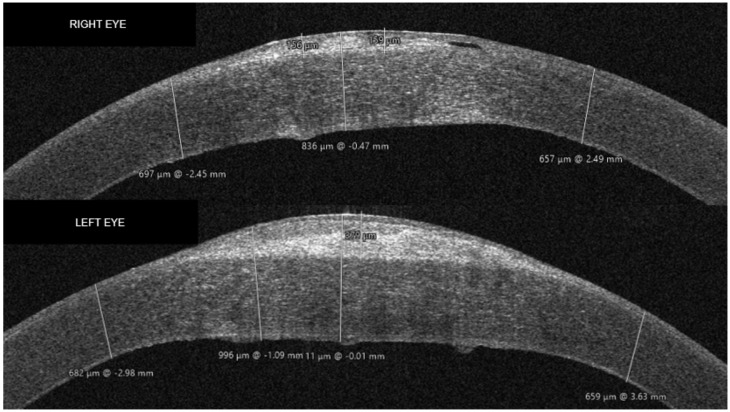
Preoperative AS-OCT aspects. Right eye (**above**): subepithelial hyperreflective deposits (~150 microns) with overlying thinned epithelium, central stromal diffuse hyperreflectivity, thickened Descemet membrane. Left eye (**below**): hyperreflective homogenous central subepithelial deposit, thin irregular overlying epithelium, Bowmann membrane discontinuities, diffuse central stromal hyperreflectivity, thickened Descemet membrane, Descemet membrane folds.

AS-OCT revealed significant differences between the RE and the LE at presentation. The LE presented a more elevated subepithelial hyperreflective deposit (~370 microns centrally), with thinned overlying epithelium, and a greater degree of Bowmann membrane discontinuity compared to the RE. The endothelial dysfunction was also more advanced in the LE, with more important central thickening of the Descemet membrane, as well as Descemetic folds, more evident centrally. The RE presented central subepithelial hyperreflective deposits (~150 microns) with thinned overlying epithelium. The DM thickening was less advanced compared to the LE. The stroma presented significant diffuse hyper-reflectivity, suggestive for stromal edema, more advanced in the LE (Figure 2).

**Figure 3:**
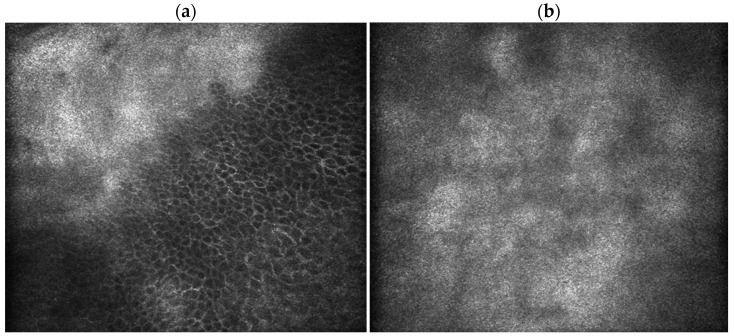

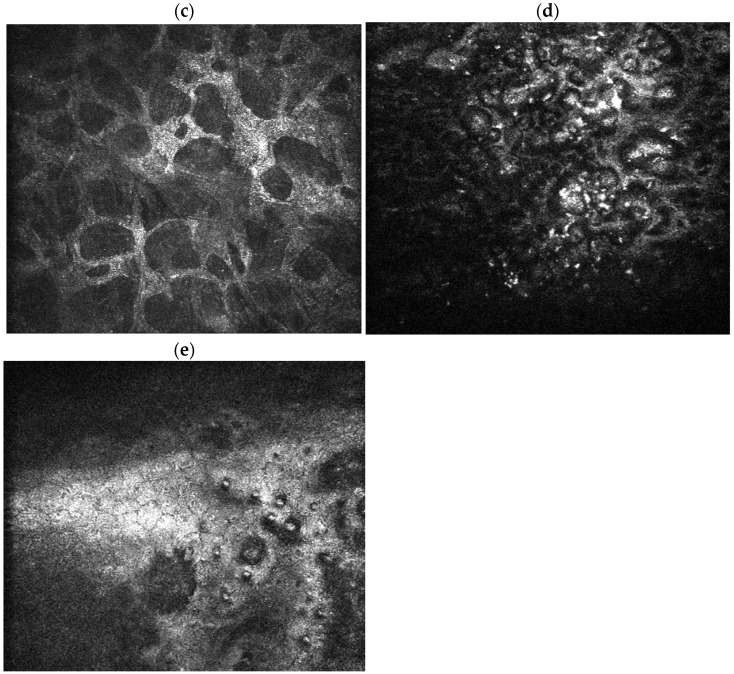
In vivo confocal microscopy (IVCM, 400 × 400 μm) of the left eye: (**a**) hyperreflective deposits interrupting the basal epithelial cell layer; (**b**) subepithelial amorphous acellular deposit; (**c**) stromal slide with reduced keratocyte density; (**d**) central endothelial slice—confluent guttae, no normal endothelial cells visible; (**e**) peripheral endothelial slice: peripheral endothelial cell mosaic exhibiting polymorphism and polymegathism near hyperreflective, round or drop-like protrusions disrupting the regular hexagonal endothelial mosaic (guttae).

**Figure 4:**
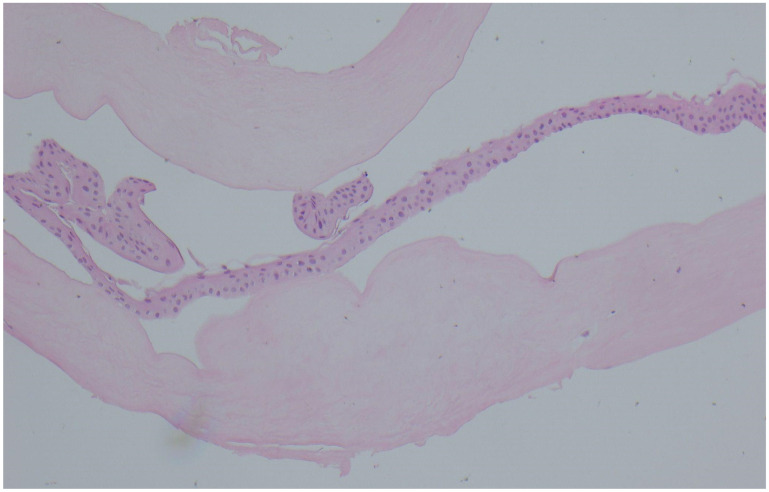
Histopathology of the excised SND (hematoxylin-eosin stain, 10× magnification). Corneal epithelial cells overlying acellular fibrous material.

IVCM identified pathognomonic features for Salzmann nodules and FECD; epithelial slices show epithelial cells interrupted by hyperreflective acellular material. The subepithelial slices show extensive acellular amorphous deposits. Endothelial slices show confluent guttae in the central area of the cornea, directly under the SND area, with no central cells with identifiable normal morphology. No endothelial cell count was available preoperatively. In the peripheral area, we identified several dispersed guttae next to EC that exhibited polymorphism (Figure 3). Other changes frequently found in FECD such as a decreased sub-basal nerve density and sparse keratocytes were also present (Figure 3). Salzmann nodular degeneration was confirmed by histopathological examination (Figure 4).

While MMP-2 quantification was not available, AS-OCT shows Bowmann membrane disruptions present in both eyes, more pronounced in the LE and irregular epithelium. These BM interruptions have been shown to favorize the deposition of subepithelial material in SND physiopathology. IVCM slices showing amorphous cellular deposits interrupting the basal epithelial cell layer, aspects confirmed by histopathology, where the deposit was identified as acellular fibrous material, consistent with other findings in the literature.

### 3.2. Surgical Management

Both eyes underwent anterior superficial keratectomy and phaco-DMEK surgery, but the timing differed as follows: RE—superficial keratectomy (March 2024), repeat superficial keratectomy for the nodule recurrence (December 2025), phaco-DMEK (December 2025). LE—superficial keratectomy (March 2024), phaco-DMEK one month after the superficial keratectomy (April 2024). As both eyes were diagnosed with advanced Fuchs endothelial dystrophy but have not undergone any prior vitrectomy and presented no extensive deep stromal fibrosis, DMEK was chosen as the preferred posterior lamellar keratoplasty technique. The DMEK graft was surgeon-prepared from fresh donor corneoscleral tissue on the day of surgery, with a graft size of 7.5 mm. In both cases, air tamponade was used. No intraoperative or postoperative complications were encountered. No rebubble was needed for either eye, as the grafts were completely attached at the 1-week post-DMEK follow-ups.

**Figure 5:**
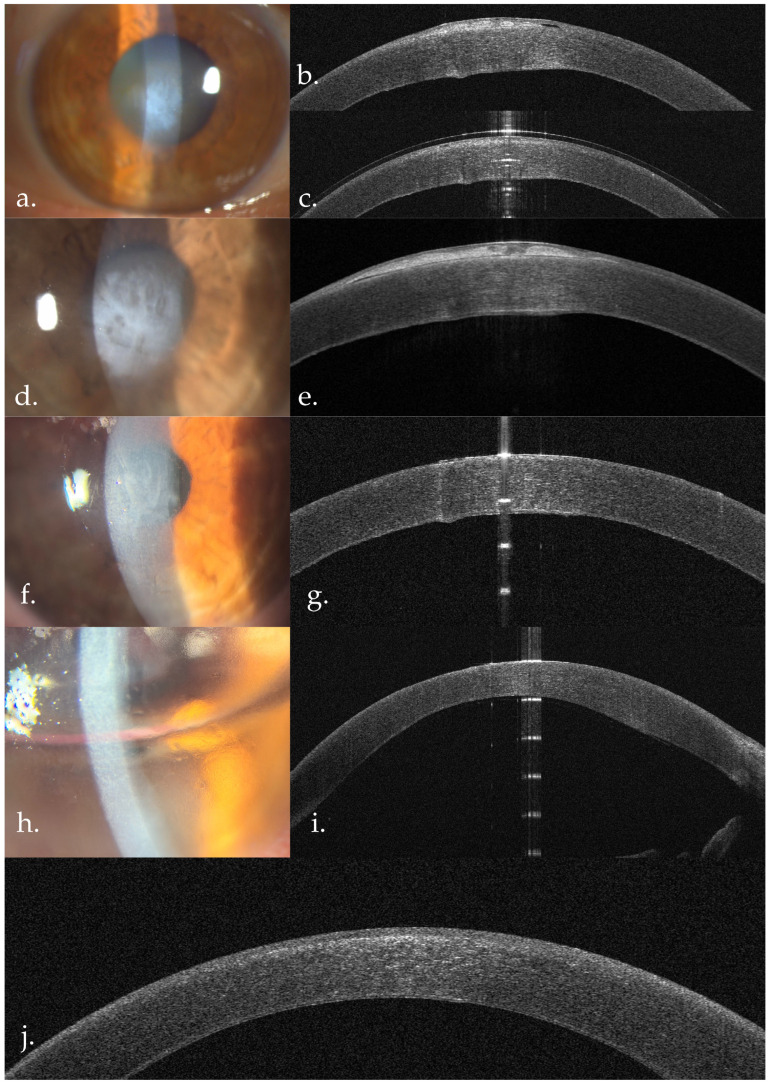
Right eye—case evolution. (**a**,**b**) Preoperative aspects. (**c**) One month after superficial keratectomy. AS-OCT: irregular epithelium overlying a hyperreflective anterior stromal area, diffuse stromal hyperreflectivity, thickened DM. (**d**,**e**) One year post keratectomy. Recurrence of the subepithelial deposits; AS-OCT: subepithelial deposits with overlying epithelium with irregular thickness, central small Bowmann membrane discontinuity; (**f**,**g**) one week post repeat superficial keratectomy; (**h**,**i**) one week after phaco-DMEK; (**j**) one month after phaco-DMEK—complete reepithelisation, discrete anterior stromal hyperreflectivity.

**Figure 6:**
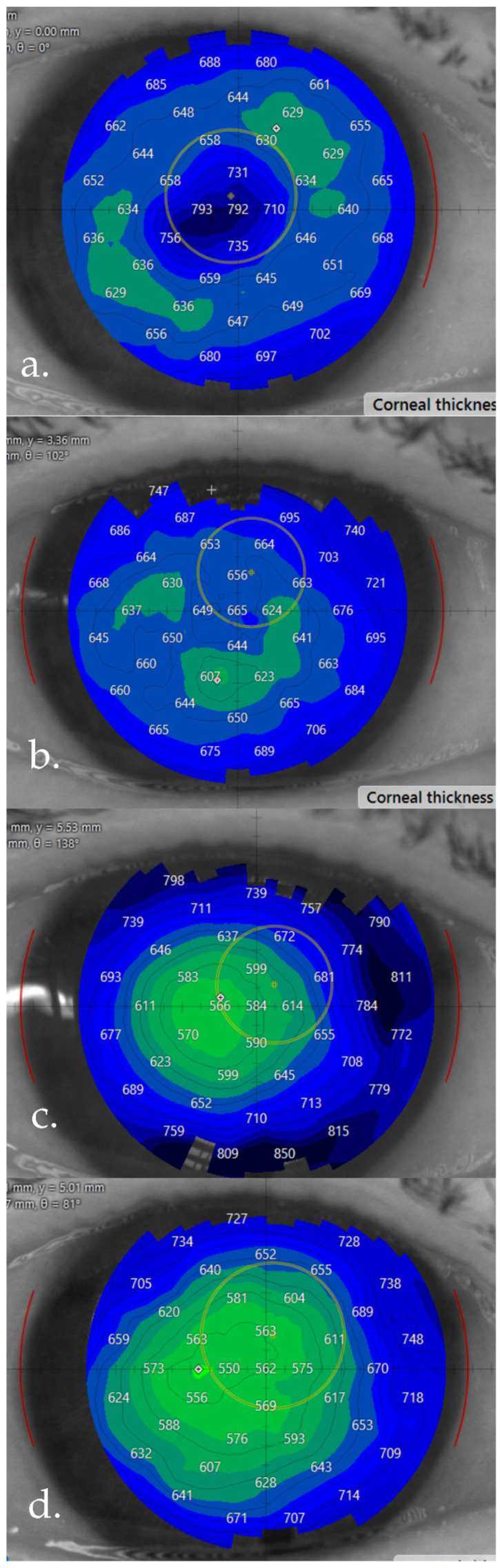
Right eye case evolution of corneal pachymetry maps. (**a**) Dec 2025—before the repeat superficial keratectomy; (**b**) one week after superficial keratectomy—isopach irregularity due to FECD is now clearly visible; (**c**) one week after phaco-DMEK; (**d**) one month after phaco-DMEK.

The first surgical procedure for the right eye was anterior superficial keratectomy (March 2024), during which the entire subepithelial nodule was removed. Preoperatively, CCT was 836 µm, out of which ~150 µm represented subepithelial deposit thickness. At the one-month follow-up in April 2024, we observed discrete Bowmann membrane irregularities, anterior stromal hyperreflectivity, but no subepithelial deposit recurrence. Central corneal thickness (CCT) measured 755 µm. At the 6-month follow-up, subepithelial deposit recurrence was observed, but no AS-OCT data was available to quantify the magnitude of the recurrence. At the 1-year follow-up (April 2025), the subepithelial deposit measured 138 µm; CCT—820 µm. At the 1.5-year follow-up, before the second keratectomy, in December 2025, the nodule measured 163 µm; CCT—792 µm. One week after the repeat keratectomy, CCT was 665 µm. Phaco-DMEK was performed. At the one-week follow-up, CCT reduced to 584 µm, and at 1 month (January 2026), 562 µm, with no subepithelial deposit recurrence (Figure 5 and Figure 6). Between the first superficial keratectomy and phaco-DMEK, the patient used preservative free artificial tears q3h, sodium chloride 5% QID, and vitamin A ointment nightly. After phaco-DMEK, the patient was prescribed a course of topical netilmicin 0.3% with dexamethasone 0.1% q6h, tapered throughout the post-operative period and long term treatment with preservative free artificial tears q6h. Preoperative, RE BCVA was 20/200, improved to 20/100 after the anterior superficial keratectomy, in April 2024, then progressively declined back to 20/200 until the repeat keratectomy (December 2025), after which the RE BCVA improved to 20/100. After the phaco-DMEK, at the one month follow-up, RE BCVA was 20/32 (Table 1). Post-operative endothelial cell count for the RE (ECD) at 1 month post-DMEK was 1377 ± 18 cells/mm^2^.

**Figure 7:**
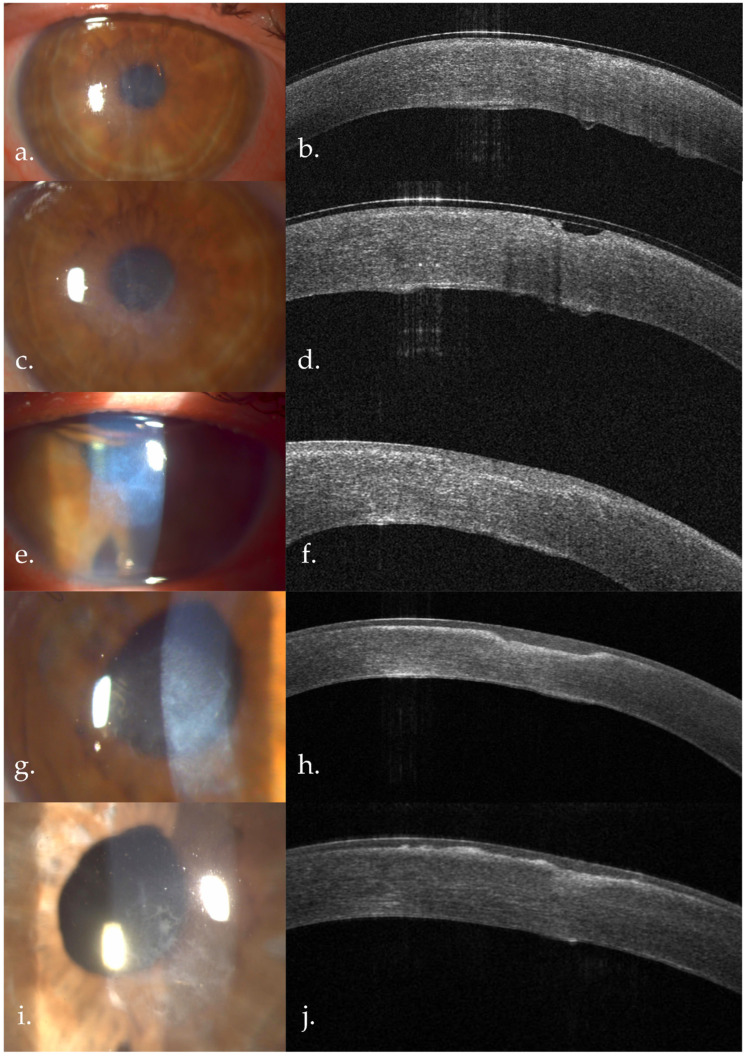
Left eye—case evolution. (**a**,**b**) One day after the superficial keratectomy. Central stromal haze, hyperreflective DM with Descemetic folds, now more clearly visible; AS-OCT: thin epithelium, Bowmann membrane defects, diffuse stromal reflectivity suggestive for edema, increased pachymetry, thickened DM, DM folds; (**c**,**d**) one month after superficial keratectomy, pre-DMEK; (**e**,**f**) one week post phaco-DMEK. AS-OCT: epithelium with varying thickness overlying the anterior stromal fibrosis, diffuse stromal hyperreflectivity, attached DMEK graft. (**g**,**h**) Six months after phaco-DMEK. More evident white-gray anterior stromal opacities. AS-OCT: important reduction of overall corneal pachymetry, anterior stromal fibrosis, reduction in overall stromal hyperreflectivity; (**i**,**j**) One year post phaco-DMEK. AS-OCT: small subepithelial deposits with slight remodeling of the anterior stromal fibrotic area.

The left eye underwent superficial anterior keratectomy in March 2024, during which the subepithelial nodule (~370 µm) was removed. Preoperative LE CCT was 1111 µm, out of which the subepithelial nodule measured ~370 µm. This procedure was done to improve visualization for the combined phaco-DMEK surgery. One week after the superficial keratectomy, directly overlying the endothelial defect, the epithelium appeared significantly thinner, with BM defect and anterior stromal hyperreflectivity. Similar aspects were seen at one month post keratectomy; CCT was 769 µm. Phaco-DMEK was done in April 2024, one month after the superficial keratectomy. One week post DMEK, corneal thickness reduced considerably (CCT—671 µm). Anterior stromal hyperreflectivity suggestive for fibrosis began to be more apparent. Six months after the DMEK, CCT was 584 µm, with anterior stromal fibrosis more evident. At the one-year follow-up, the stromal reflectivity overall became more homogenous, with discrete subepithelial deposits (~37 µm) (Figure 7). Postoperative LE ECD at 1.5 years after DMEK was 979 ± 13 cells/mm^2^. LE BCVA was 20/400 preoperatively in March 2024, improved to 20/100 after the superficial keratectomy (April 2024), an steadily improved afterwards: 1 week post phaco-DMEK—20/70, and stabilized at 20/32 after the 6 month follow-up (Table 2). Medical management was a course of topical netilmicin 0.3% with dexamethasone 0.1% q6h, tapered throughout the post-operative period and long-term treatment with preservative free artificial tears q6h.

## 4. Discussion

FECD is classified as a dystrophy of the corneal endothelium [11]. As the disease progresses, secondary to the endothelial layer dysfunction, FECD determines a series of morphological and functional alterations to the other corneal layers [14]. Clinically, one can observe increased Descemet membrane thickness with a beaten bronze appearance, guttae, stromal haze and sometimes epithelial dysfunction, with corneal erosions [17]. IVCM studies have identified changes that start even from the early stages of FECD, such as reduced sub-basal nerve density, increased epithelial dendritic cell count [12,15], stromal changes such as anterior stromal keratocyte depletion [13]. We found similar aspects at the IVCM examination in the present study, in both eyes, with very sparse sub-basal nerves, barely visible. The subepithelial slices of the central cornea showed extensive amorphous acellular deposits, consistent with the histopathological findings.

Regarding the pathophysiological processes involved in SND, it has been identified that the central epithelial cells in the SND area express a series of markers usually found in the stem cells in the limbal area, suggesting increased metabolic activity. While the normal epithelium expresses factors like matrix-metalloproteinases (MMP), TGF-β1 and platelet-derived growth factor (PDGF), the stromal cells are not affected, the epithelial basement membrane (EBM) acting as a barrier [18]. This barrier seems to be disrupted by the upregulation of MMP-2 that affects the primary component of the EBM, type IV collagen, leading to dysfunction both in the EBM and in the Bowmann layer [2,19]. In a study by Stone et al., immunohistochemical staining has identified MMP-2 in the overlying epithelium and vimentin in the stroma in all studied samples. These disruptions offer a passage for the cytokines to the keratocytes that migrate anteriorly and differentiate into fibroblasts/myofibroblasts, producing subepithelial fibrotic extracellular matrix (ECM) that constitutes the nodules [19].

While in our case MMP-2 quantification was not available, AS-OCT shows Bowmann membrane disruptions present in both eyes, more pronounced in the LE and irregular epithelium. These BM interruptions have been shown to favorize the deposition of subepithelial material in SND physiopathology. IVCM slices showing amorphous cellular deposits interrupting the basal epithelial cell layer, aspects confirmed by histopathology, where the deposit was identified as acellular fibrous material, consistent with other findings in the literature [2].

Regarding the medical and surgical approach in SND, first-line therapy is based on a conservative approach: intensive lubrication, lid hygiene, warm compresses, and short courses of topical corticosteroids for symptomatic flares; oral doxycycline may be added where evaporative dry eye or blepharitis is prominent [2]. The indications for surgical treatment include visually significant irregular astigmatism, central nodules, and failure of conservative care. Superficial keratectomy and excimer laser phototherapeutic keratectomy are the most commonly used techniques. Anterior lamellar keratoplasty is reserved for advanced cases with significant stromal opacities [20,21].

While frequently, epithelial and Bowmann membrane injuries heal with Bowmann membrane defects and anterior stromal scarring, in our case the epithelial injury seems to have favored the development of SND. While usually the Salzmann nodules interest the corneal periphery [5], in our case the patient never had any peripheral SND lesions, nor did she develop additional lesions in the peripheral cornea at any point. From the debut of the disease, both nodules were present directly over the area most affected by FECD.

The subepithelial nodules created severely altered pachymetry maps, which made the grading of the corneal dysfunction difficult. After the nodule removal, we found important loss of isopath regularity, posterior surface depression and displacement of corneal thinnest point, consistent with advanced FECD according to new criteria described by Yasukura et al. and Patel SV et al. [22,23].

After the anterior superficial keratectomy, visualization was good enough to perform combined cataract surgery and posterior lamellar keratoplasty. We decided to wait one month after the initial keratectomy involving the left eye in order for corneal reepithelization to occur and get more accurate biometry data. Posterior lamellar keratoplasty, particularly the DMEK technique was used both due to surgeon preference and due to shorter recovery times compared to penetrating keratoplasty, more predictable post-operative refractive outcomes and a lower rejection rate [24]. In our patient, the main cause of decreased visual acuity after the nodule removal was due to the stromal edema. While stromal fibrosis can be a contraindication for posterior lamellar keratoplasty, in our case the fibrosis only involved the anterior stroma. We noted the absence of deep stromal fibrosis, as well as preDescemetic fibrosis, which could lower postoperative visual recovery. Posterior stromal ripples, which can be a risk factor for early graft detachment [25], were observed, however postoperatively our patient needed no rebubble procedures in either eye, as the graft was completely attached.

After the superficial keratectomy and DMEK, the area with the Bowmann defect presented a thin layer of fibrosis in the anterior stroma, with little signs of progression. The right eye, which underwent superficial keratectomy during the same period of time, showed important nodular growth during the follow-up period. The post-operative evolution was especially interesting, due to the fact that it provided insights into the different rates of recurrence of the SND nodules between the LE that underwent superficial keratectomy and DMEK one month apart, and the RE, for which the first superficial keratectomy and DMEK was almost two years after, showing a possible link between the central corneal area which was most affected by FECD, which possibly triggered an abnormal healing response with led to the development of SND. Therefore, in this case we believe that removing the cause of corneal dysfunction significantly reduced the magnitude of recurrence of the SND.

To our knowledge, so far our report is the first case of bilateral central extensive SND associated with FECD, in the context of no other present ocular or systemic pathologies. Furthermore, we present a unique perspective into how the endothelial dysfunction due to FECD affects the entire cornea, ushering in changes that ultimately affect even the Bowmann membrane and epithelial layer. Slit-lamp, AS-OCT and IVCM findings confirm the FECD diagnosis.

## 5. Conclusions

With this case report, we aim to shine the spotlight on a rare, but important way in which FECD can impact the cornea beyond the “classic” corneal edema typically associated with advanced FECD. The impact of the endothelial dysfunction on the pathophysiological processes that in our case seem to favor the formation of SND are incompletely understood, further research being needed. Furthermore, we aim to bring attention to how restoring the endothelial function with the DMEK shaped the corneal remodeling in our case. Further research is needed to better understand the association between FECD and SND.

## Figures and Tables

**Table 1:** Clinical evolution of the right eye.

	**Preoperative (March 2024)**	**Superficial Anterior Keratectomy**	**1 Month After Superficial Keratectomy (April 2024)**	**1 Year After Superficial Keratectomy (April 2025)**	**December 2025—Before 2nd Superficial Keratectomy**	**Phaco-DMEK**	**1 Week Post Superficial Keratectomy (December 2025)**	**1 Week After DMEK (December 2025)**	**1 Month After DMEK (January 2026)**
BCVA RE	20/200		20/100	20/200	20/200		20/100	20/70	20/32
CCT RE (µm)	836		755	820	792		665	584	562
Subepithelial deposit thickness (µm)	156		<10 µm	138	163		none	none	none
HOA 3 mm (µm)	n/a		n/a	4.04 µm	2.25 µm		0.96 µm	0.18 µm	0.20 µm

**Table 2:** Clinical evolution of the left eye.

	**Preoperative (March 2024)**	**Superficial Anterior Keratectomy**	**1 Month After Superficial Keratectomy (April 2024)**	**Phaco-DMEK**	**1 Week After DMEK (April 2024)**	**6 Months After DMEK**	**1 Year After DMEK (April 2025)**	**1.5 Years After DMEK (December 2025)**
BCVA left eye	20/400		20/100		20/70	20/32	20/32	20/32
CCT LE (µm)	1111		769		671	584	576	610
Subepithelial deposit thickness (µm)	379		none		none	none	37	37
HOA 3 mm (µm)	n/a		n/a		1.25 µm	1.26 µm	1.12 µm	0.80 µm

## Data Availability

The original contributions presented in this study are included in the article. Further inquiries can be directed to the corresponding author(s).

## Abbreviations

The following abbreviations are used in this manuscript:

AS-OCT	anterior segment optical coherence tomography
BCVA	best-corrected visual acuity
DMEK	Descemet membrane endothelial keratoplasty
FECD	Fuchs endothelial corneal dystrophy
HOA	high order aberrations
HSV	herpes simplex virus
IVCM	in vivo confocal microscopy
LE	left eye
SND	Salzmann nodular degeneration
RE	right eye
